# Letter from Prof. William James, Harvard University, Cambridge, Mass

**Published:** 1890-07

**Authors:** William James

**Affiliations:** Harvard University, Cambridge, Mass.


From Prof. WILLIAM JAMES,
Harvard University, Cambridge, Mass.

*To the Editor of Atlanta Medical and Surgical Journal:*

DEAR SIR—May I ask for the publicity of your pages to aid me in procuring co-operation in a scientific investigation for which I am responsible? I refer to the *Census of Hallucinations*, which was begun several years ago by the "Society for Psychical Research," and of which the International Congress of Experimental Physiology at Paris, last summer, assumed the future responsibility, naming a committee in each country to carry on the work.

The object of the inquiry is two-fold: 1st, to get a mass of facts about hallucinations which may serve as a basis for a scientific study of these phenomena; and 2d, to ascertain approximately the *proportion of persons* who have had such experiences. Until the average frequency of hallucinations in the community is known, it can never be decided whether the so-called "veridical" hallucinations (visions or other "warnings" of the death, etc., of people at a distance) which are so frequently reported, are accidental coincidences, or something more.

Some 8,000 or more persons in England, France and the United States have already returned answers to the question which heads the census sheets, and which runs as follows:

"*Have you ever, when completely awake, hnd a vivid impression of seeing or being touched by a living being or inanimate object, or of hearing a voice, which impression, so far as you could discover, was not due to any external physical cause?*"

The "Congress" hopes that at its next meeting, in England in 1892, as many as 60,000 answers may have been collected It is obvious that for the purely statistical inquiry, *the answer "No" is as important as the answer "Yes."*

I have been appointed to superintend the Census in America, and I most earnestly bespeak the co-operation of any among your readers who may be actively interested in the subject. It is clear that very many volunteer canvassers will be needed to secure success. Each census blank contains instructions to the collector, and places for twenty-five names; and special blanks for the "Yes" cases are furnished in addition. I shall be most happy to supply these blanks to any one who will be good enough to make application for them to

Yours truly,

(Professor) WM. JAMES,

Harvard University, Cambridge, Mass.

